# High-contrast and fast electrochromic switching enabled by plasmonics

**DOI:** 10.1038/ncomms10479

**Published:** 2016-01-27

**Authors:** Ting Xu, Erich C. Walter, Amit Agrawal, Christopher Bohn, Jeyavel Velmurugan, Wenqi Zhu, Henri J. Lezec, A. Alec Talin

**Affiliations:** 1 National Laboratory of Solid State Microstructures, College of Engineering and Applied Sciences and Collaborative Innovation Center of Advanced Microstructures, Nanjing University, 22 Hankou Road, Nanjing 210093, China; 2 Center for Nanoscale Science and Technology, National Institute of Standards and Technology, Gaithersburg, Maryland 20899, USA; 3 Maryland Nanocenter, University of Maryland, College Park, Maryland 20742, USA; 4 Sandia National Laboratories, Livermore, California 94551, USA

## Abstract

With vibrant colours and simple, room-temperature processing methods, electrochromic polymers have attracted attention as active materials for flexible, low-power-consuming devices. However, slow switching speeds in devices realized to date, as well as the complexity of having to combine several distinct polymers to achieve a full-colour gamut, have limited electrochromic materials to niche applications. Here we achieve fast, high-contrast electrochromic switching by significantly enhancing the interaction of light—propagating as deep-subwavelength-confined surface plasmon polaritons through arrays of metallic nanoslits, with an electrochromic polymer—present as an ultra-thin coating on the slit sidewalls. The switchable configuration retains the short temporal charge-diffusion characteristics of thin electrochromic films, while maintaining the high optical contrast associated with thicker electrochromic coatings. We further demonstrate that by controlling the pitch of the nanoslit arrays, it is possible to achieve a full-colour response with high contrast and fast switching speeds, while relying on just one electrochromic polymer.

## Abstract (web summary)

Slow switching speeds in device configurations have severely limited the applications of electrochromic materials. Here, Xu *et al*. use plasmonic nanoslit arrays and demonstrate fast, high-contrast, monochromatic and full-colour electrochromic switching using two different electrochromic polymers.

## Graphical abstract

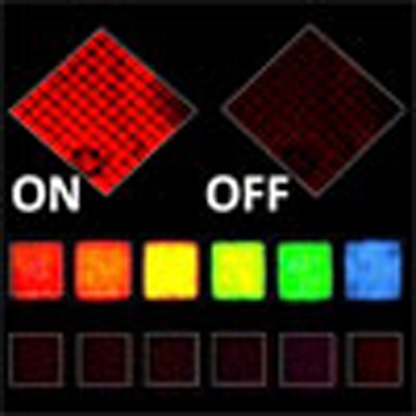

Advances in flat-panel display technology over the last-decade have enabled a new generation of portable electronic equipment. Ultra-thin, flexible and low-power-consuming displays drive the rapid growth in electronic readers, reconfigurable signage and a host of other consumer electronics. Current mainstream commercial devices depend on electrophoretic displays (EPDs)^1^, liquid crystal displays^2^ and organic light-emitting diodes^3^. EPDs, collectively known as electronic ink, provide very sharp images, bistability and excellent outdoor readability. However, EPDs have limited potential for displaying full-colour images, and their response time and refresh rate are too slow for sophisticated interactive applications and video. Although liquid crystal displays and organic light-emitting diodes do enjoy full-colour scheme and shorter response time, they are not bistable, and their high-cost and complex manufacture remain a challenge. Other emerging display technologies based on photonic crystals^4^,^5^,^6^ and quantum dots^7^,^8^,^9^ still require significant development.

Discovered in the late 1960s, electrochromic materials, including various transition metal oxides and conducting polymers, show a reversible colour change on electrochemical reduction or oxidation by application of a small voltage^10^. Electrochromic materials are particularly attractive for flexible display applications because of their vibrant colours, low-cost and relatively simple processing requirements^11^,^12^,^13^,^14^. Nevertheless, long switching times, typically on the order of seconds, have limited electrochromic materials to niche applications^15^. In general, the switching time *τ* of electrochromic devices is limited by ionic diffusion in the electrochromic material (*τ*∝*L*^2^/*D*, where *L* is the film thickness and *D* is the diffusivity). Switching times on the order of tens of milliseconds have been demonstrated for certain multilayer-thin electrochromic polymer films, although at a cost of substantially reduced optical contrast, in general <30% (ref. 16). In addition to speed, a typical multi-colour electrochromic display requires at least three separate electrochromic materials to provide the colours necessary to make an additive, red–green–blue, or a subtractive, cyan–magenta–yellow, colour palette. These three separate layers can require up to six layers of transparent conductors to operate, raising production complexity, cost and further limiting the switching contrast.

With recent advancements in nanofabrication and optical characterization techniques, surface plasmon polariton (SPP)-based nanoscale photonic and optoelectronic devices have generated considerable interest^17^. SPPs are photon-induced collective charge oscillations that are able to sustain the propagation of optical frequency electromagnetic waves at an interface between a dielectric medium and a metal. The tight spatial confinement and high local field intensity associated with SPPs have enabled operation of nano-optical devices beyond the optical diffraction limit^18^. More recently, plasmonic nanostructures, such as metallic nanohole arrays and nanoparticles, have been used to explore electro-optic switching^19^ and electrochemical tunability of localized surface plasmon resonances^20^,^21^,^22^.

Here, using plasmonic nanoslit arrays, we demonstrate high contrast, fast monochromatic and full-colour electrochromic switching using two different electrochromic polymers, polyaniline (PANI) and poly(2,2-dimethyl-3,4 propylenedioxythiophene) (PolyProDOT-Me_2_). Unlike transition-metal-oxide electrochromic materials, which are usually sputter coated, or inorganic polymers such as Prussian blue, which often form nanocrystals and are therefore difficult to deposit uniformly over high-aspect ratio features^23^, both PANI and PolyProDOT-Me_2_ polymers can be electrodeposited as conformal, extremely thin coatings on metal structures with well-controlled thicknesses^24^,^25^, favouring scatter-free propagation of SPPs with maximum interaction with the electrochromic films. As a result, the plasmonic electrochromic switchable configurations retain the advantages of both fast switching speed and high optical contrast.

## Results

### Plasmon-enhanced monochromatic electrochromic switching

**Figure 1:**
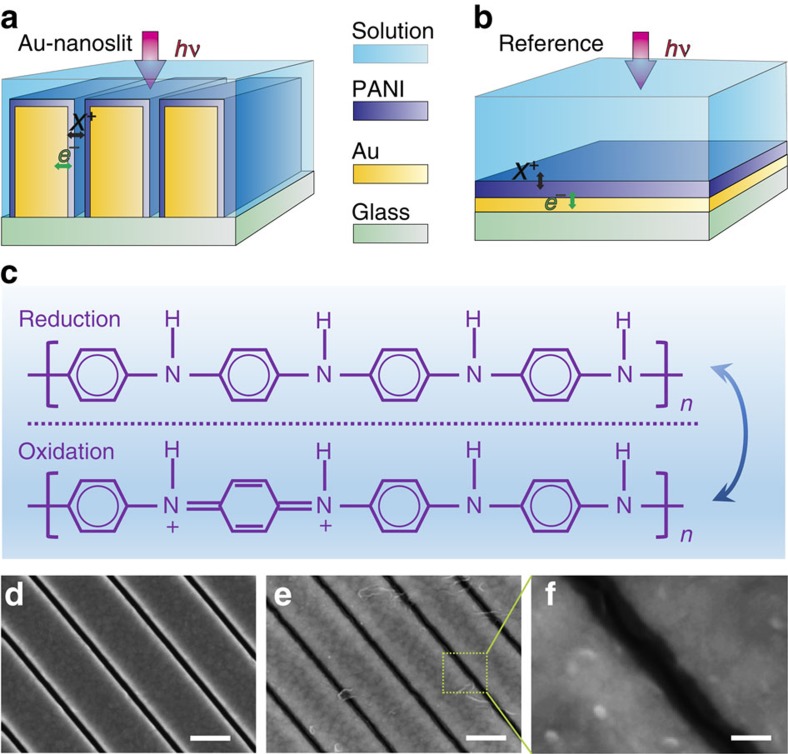
Plasmonic electrochromic electrodes. Schematic diagram of a plasmonic electrochromic electrode incorporating (**a**) Au-nanoslit array and (**b**) reference planar electrochromic electrode. The pitch of the Au-nanoslit array is 500 nm. The depth and width of the slit is 60 and 250 nm, respectively. (**c**) Chemical structures of PANI in the reduced and oxidized form. SEM images of the fabricated Au-nanoslit electrode (**d**) before and (**e**) after deposition of a PANI to a thickness *d*≈15 nm. (**f**) Magnified SEM image from **e**. Scale bars, 300 nm (**d**,**e**). Scale bar, 100 nm (**f**).

The working electrode designed for monochromatic operation incorporates an Au film patterned with a nanoslit array and conformally coated with a thin layer of PANI (‘Au-nanoslit', Fig. 1a). The Au-nanoslit electrode is immersed in an electrolyte solution, along with a Pt counter electrode and reference electrode. A voltage applied to the working electrode causes electrons (from the metal) and ions (from the electrolyte) to either flow in (reduction) or out (oxidation) of the polymer, thus changing its state of charge and, concurrently, its optical absorption characteristics^26^. Light normally incident on the Au-nanoslit array couples to SPPs travelling both as surface waves along the illuminated Au surface and as guided modes in the nanoslits, with field maxima occurring at the Au-polymer interfaces. The electrochromic material's effective optical thickness for transmission is thus close to that of the slit depth, which can far exceed the physical thickness of the electrochromic layer. Therefore, strong optical absorption can be realized using a thin electrochromic polymer layer, while simultaneously achieving a fast switching time due to correspondingly small charge-propagation distance (which is normal to the film surface and orthogonal to the direction of light propagation). This combination of efficient optical modulation and switching speed cannot be achieved with a more conventional planar, unpatterned configuration provided by an equally thin electrochromic polymer film coated on a semi-transparent thin Au film illuminated at normal incidence (‘Reference', Fig. 1b). Achieving an effective electrochromic optical thickness and switching contrast comparable to that of the plasmonic nanoslit structure implies the use of a much thicker polymer layer, leading to longer charge diffusion distances and slower switching speeds. The chemical structures of PANI in the oxidized and reduced form are shown in Fig. 1c.

Patterned electrodes corresponding to the Au-nanoslit geometry are fabricated by sputter deposition of a 250-nm-thick Au film onto a 25 mm × 25 mm borosilicate glass substrate pre-coated with a 5-nm-thick Ti adhesion layer, followed by focused ion beam (FIB) milling of a nanoslit array (nominal slit width *w*=60 nm; pitch *P*=500 nm) over a 10 μm × 10 μm area. Scanning electron microscope (SEM) images of the resulting array, before and after deposition of PANI by potentio-dynamic cycling to a thickness *d*≈15 nm, are shown in Fig. 1d–f. Other Au-nanoslit arrays are coated with similarly prepared PANI films of thicknesses *d* ranging from ≈5 nm to ≈25 nm. Reference planar electrodes are also fabricated by depositing PANI films under identical conditions to those of Au-nanoslit electrodes on glass substrates coated with 25-nm-thick Au films. A custom-built photoelectrochemical cell is used to switch the PANI films between the reduced form (‘ON' state, applied voltage *V*_ON_=−0.2 V versus Ag/AgCl) and oxidized form (‘OFF' state, applied voltage *V*_OFF_=0.3 V versus Ag/AgCl). Within these potential limits, the PANI films are not further oxidized to the emeraldine base or the pernigraniline states, to avoid polymer degradation over thousands of switching cycles. The transmission spectra of PANI film with different applied voltages are shown in Supplementary Fig. 1. A HeNe laser working at *λ*=632.8 nm in transverse-magnetic (TM) polarization (electric field perpendicular to the slit length) is used to illuminate both Au-nanoslit and reference planar electrodes at normal incidence, while the polymer is cycled between its clear (‘ON') and absorbing (‘OFF') states. The transmitted light is collected using an inverted optical microscope and its intensity is measured using a Si-photodiode connected to the output ports of the microscope.

**Figure 2:**
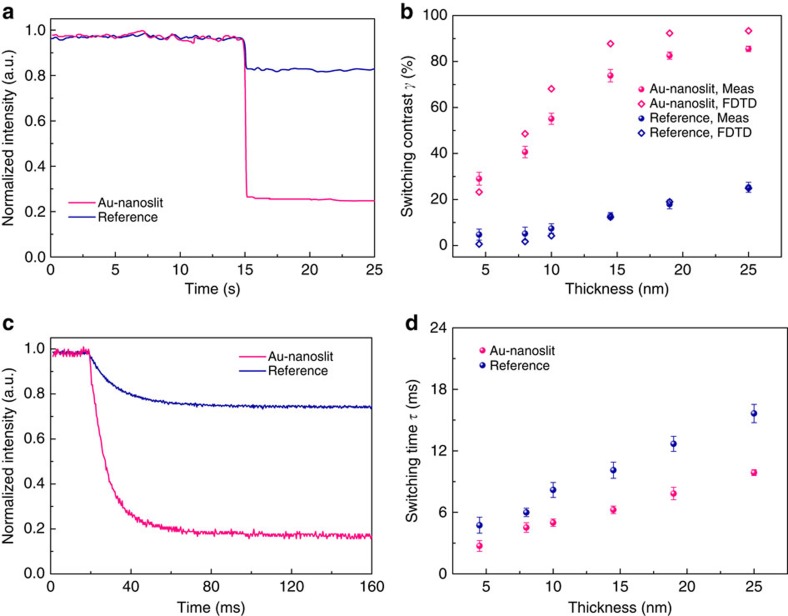
Experimental results for Au-nanoslit and reference planar electrodes. (**a**) Transmitted light intensity as a function of time for, respectively, Au-nanoslit and reference planar electrodes coated with 25 nm-thick PANI layer, given a step transition in applied voltage at *t*=15 s, from −0.2 V (clear) to 0.3 V (absorbing) state. (**b**) Experimentally measured and numerically simulated switching contrast *γ* as a function of PANI thickness for Au-nanoslit and reference planar electrodes. The refractive indices of PANI film in clear and oxidized forms at different thickness used in simulations are taken from ref. 21 and fitted by Boltzmann and exponential functions. Error bars, s.d. for repeated experimental measurements (four in total). (**c**) Transmitted light intensity (measured using photodiode) as a function of time for Au-nanoslit and reference planar electrodes, each coated with 25-nm-thick PANI films. (**d**) Switching time *τ* for Au-nanoslit and reference planar electrodes as a function of PANI film thickness. Error bars, s.d. for repeated measurements (four in total).

In Fig. 2a, we show the transmitted light intensity (*I*) versus time (*t*) at *λ*=632.8 nm for the Au-nanoslit and reference planar electrodes, where an abrupt step transition in the applied voltage from −0.2 V versus Ag/AgCl to 0.3 V versus Ag/AgCl is applied at time *t*=15 s. Both electrodes are coated with a PANI film of thickness *d*≈25 nm. As expected, the Au-nanoslit electrode exhibits significantly higher optical modulation amplitude than the reference planar one. The absolute optical transmission through the Au-nanoslit electrode at wavelength of 632.8 nm in the reduced state is ≈10 %. Figure 2b plots the optical switching contrast, defined as *γ*=(*I*_ON_−*I*_OFF_)/*I*_ON_, as a function of the PANI film thickness *d* for various Au-nanoslit and reference planar electrodes, where *I*_ON_ and *I*_OFF_ refer to, respectively, the transmitted intensity in the reduced and oxidized form of the PANI films. In the case of the Au-nanoslit geometry, *γ* monotonically increases as a function of film thickness, linearly at smaller thicknesses and with a noticeable roll off beyond *d*=15 nm, owing to the onset of negligible transmission in the OFF state as a result of near-full absorption by the polymer fully filling the slits. These trends are substantiated by finite-difference-time-domain simulations replicating the light transmission through PANI-coated Au-nanoslit and reference electrodes, using the published refractive index values of as-deposited PANI films measured by *in-situ* ellipsometry^27^. Simulations for a given transmission state and under identical illumination conditions (Supplementary Fig. 2) indicate that the light intensity is significantly enhanced within the PANI films coating the slit sidewalls of the Au-nanoslit structure, relative to the case of the PANI film coating the planar surface of the reference structure, resulting in a higher optical modulation efficiency per unit interaction length.

Figure 2c displays the temporal switching characteristics of the Au-nanoslit and unpatterned reference electrodes, each coated with PANI films of nominal thickness 25 nm. The Au-nanoslit electrode exhibits a faster switching time (*τ*≈9 ms) compared with that of the reference electrode (*τ*≈14 ms). The switching time *τ* is calculated by fitting a decaying-exponential function to the transmitted intensity in each case using the expression *I*(*t*)=*A*+*γ*exp(*−t*/*τ*), where *I*(0)=*A*+*γ*=1 is the normalized intensity as measured on the photodiode at ON state, *A* is the steady-state normalized intensity at OFF state (*t*>>*τ*) and *γ* is the switching contrast. Figure 2d summarizes the measured switching time, *τ*, for various Au-nanoslit and reference electrodes plotted as a function of the PANI film thickness. For any given PANI film thickness, the switching times for the Au-nanoslit electrodes are unexpectedly lower than those of the corresponding reference ones. The lower values of *τ* for the Au-nanoslit electrodes possibly result from polymer deposition on the slit sidewalls to values that are lower than the nominal value assumed for deposition on a planar surface.

The ratio of optical contrast to the switching time, *γ*/*τ*, plotted in Supplementary Fig. 3, defines a useful figure-of-merit (FOM) for electrochromic switching. The experimental FOM values for the Au-nanoslit electrode are approximately one order of magnitude higher than those of the corresponding unpatterned reference electrode over the explored PANI film thickness range *d*≈5 nm to *d*≈25 nm, achieving a maximum for *d*≈15 nm. Conversely, the corresponding FOM for the reference planar electrode increases monotonically over the same thickness range. Supplementary Fig. 4a displays an SEM image of a large-area Au-nanoslit electrode composed of a 10 × 10 matrix of individual nanoslit arrays (each 10 μm × 10 μm in area), coated with a 15-nm-thick PANI layer. Transmission of red light (*λ*=632.8 nm) through the electrodes in the ON state (applied voltage *V*_ON_=−0.2 V versus Ag/AgCl) and OFF state (applied voltage *V*_OFF_=0.3 V versus Ag/AgCl) is illustrated in Supplementary Fig. 4b,c, respectively. Real-time switching of red light transmitted through the Au-nanoslit electrode as the applied voltage is repeatedly stepped between *V*_ON_ and *V*_OFF_ is demonstrated in Supplementary Movie 1. These results summarize the high optical contrast and fast switching speed associated with the plasmonic Au-nanoslit electrode designed for monochromatic operation in the red.

### Plasmon-enhanced full-colour electrochromic switching

In addition to the monochromatic switching, we fabricate another set of plasmonic electrodes using a hybrid geometry including a nitride planar waveguide, a slit-patterned Al metal substrate and an electrochromic polymer coating, to demonstrate full-colour and fast switching capability across the entire visible range. It has been shown that nanoslit arrays fabricated with deep-subwavelength slit widths on opaque metal films can function as spectral filters^28^,^29^,^30^,^31^,^32^,^33^ and their peak spectral transmission can be tuned by altering the period of the array. Efficient visible-frequency operation of the Au-nanoslit device described in the previous section is limited to the red spectral range, because SPP propagation losses along Au surfaces are higher at shorter wavelengths^34^. Furthermore, the high cost of Au makes it impractical for use in consumer display applications. To overcome these challenges, we use a modified plasmonic device geometry leveraging Al coated with a thin layer of PolyProDOT-Me_2_, to achieve a full-colour electrochromic optical response. We use Al because it is a low-cost, earth-abundant metal that supports SPPs with low optical losses in the ultraviolet and visible regions of the spectrum. In addition, previous work on plasmonic devices fabricated using Al also confirms that this metal is a good candidate for SPP-based colour filters^29^,^30^,^31^. Furthermore, we use PolyProDOT-Me_2_ because it is a high-colouration-efficiency electrochromic polymer that exhibits broadband optical absorption^35^. The peak absorbance of the PolyProDOT-Me_2_ lies in the centre of visible spectrum (*λ*_peak_≈570 nm), making it suitable for fast switching applications across the entire visible range. Finally, the electrodeposition and electrochemical cycling conditions for PolyProDOT-Me_2_ are completely compatible with Al.

**Figure 3:**
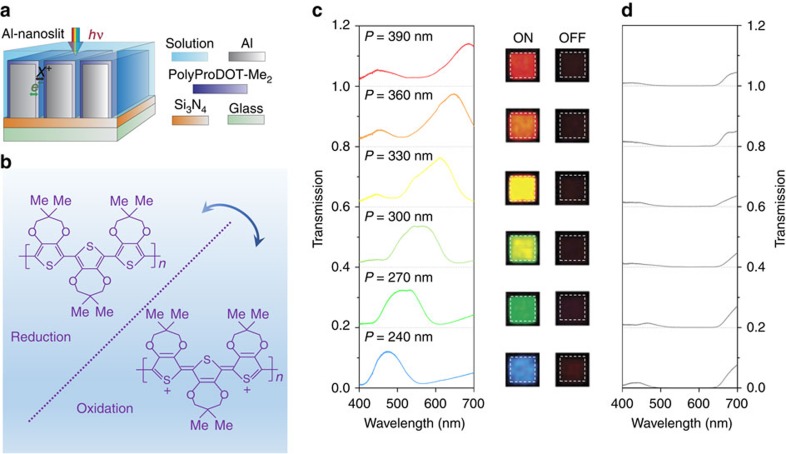
Full-colour plasmonic electrochromic electrodes. (**a**) Schematic diagram of a plasmonic electrochromic electrode incorporating Al-nanoslit array. The pitch of six Al-nanoslit arrays ranges from 240 to 390 nm, as a step of 30 nm. The thickness of Al layer and Si_3_N_4_ waveguide layer is 250 and 170 nm, respectively. (**b**) Chemical structures of PolyProDOT-Me_2_ in the oxidized and reduced form. (**c**,**d**) Optical transmission spectra of PolyProDOT-Me_2_-coated Al-nanoslit structures with respective values of slit period *P*=240, 270, 300, 330, 360 and 390 nm, along with corresponding optical micrographs of device areas imaged in transmission. Transmission spectra and micrographs for (**c**) ON and (**d**) OFF states of the polymer are displayed, respectively.

The schematic diagram of the Al-nanoslit electrode is shown in Fig. 3a. In contrast to the monochromatic Au-nanoslit structure described earlier, a Si_3_N_4_ waveguide is added as a buffer layer underneath the Al nanoslit array to further narrow the filtered spectral linewidth and increase its colour purity^32^. Al-nanoslit electrodes of various slit-array periods *P* are prepared via physical vapour deposition of a 170-nm-thick Si_3_N_4_ waveguide layer on a 25 mm × 25 mm borosilicate glass substrate, followed by sputter deposition of a 250-nm-thick Al film. Nanoslit arrays with nominal slit width ≈70 nm wide are then patterned through the Al using FIB milling to form multiple slit arrays each covering areas of 10 μm × 10 μm, with *P* ranging from 240 to 390 nm in steps of 30 nm. The thin native oxide that readily forms on the Al surface can block charge flow necessary for electrodeposition (as well as for subsequent electrochemical cycling); to inhibit formation of such an insulating layer, a 4-nm-thick conformal coating of Pt is deposited using atomic layer deposition, before polymer electrodeposition. Finally, a 15-nm-thick layer of PolyProDOT-Me_2_ is electrodeposited onto the surface, conformally coating the nanoslit arrays. The chemical structures of PolyProDOT-Me_2_ in the oxidized and reduced form are shown in Fig. 3b.

In Fig. 3c,d, we show the experimentally measured optical transmission spectra of the fabricated Al-nanoslit electrodes, along with corresponding optical micrographs, for both transmitting ON (applied voltage *V*_ON_=0.2 V versus Ag wire) and absorbing OFF (applied voltage *V*_OFF_=−0.6 V versus Ag wire) states of the polymer. In contrast to the uniformly dark colours exhibited in the OFF state, the Al-nanoslit electrodes in the ON state show, as a function of period, an assortment of vivid colours covering the entire visible spectrum. The experimentally measured absolute transmission at filtered wavelengths in the ON state ranges from 13 to 18%. The switching contrast of each Al-nanoslit electrode averaged over the entire visible spectrum, 
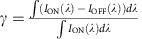
, ranges from 73 to 90%. The experimentally measured switching time for the Al-nanoslit electrode is *τ*≈80 ms. We expect that optimization of the photoelectrochemical cell, such as decreasing the non-patterned electrode area (thus, decreasing the cell capacitance) and decreasing electrolyte resistance will further decrease the switching time.

## Discussion

The switching speed of the plasmonic electrochromic electrodes demonstrated here, on the order of tens of milliseconds, is comparable to pixel switching speeds in commercial displays and is compatible for use in sophisticated applications requiring dynamic switching. Although the On-state (reduced state) absolute transmission efficiency of the electrodes are lower than those of commercial liquid crystal pixels, we expect that it can be further improved by optimizing plasmonic nanostructures or using more efficient polymers. The plasmonic electrochromic electrodes implemented here, each only tens of micrometres in lateral dimension, provide a potential pathway for achieving switchable pixels that are approximately one to two orders of magnitude smaller than those used in current high-definition displays^36^. In particular, the Al-based plasmonic electrochromic electrodes display high contrast and rapid modulation over the full-colour gamut using only one electrochromic polymer. Circumventing the need for multiple electrochromic polymers avoids potential material incompatibilities and considerably simplifies the fabrication process while reducing manufacturing costs.

In conclusion, Au and Al metallic nanoslit arrays conformally coated with electrochromic polymers are shown to enhance the optical performance of the polymer materials, yielding the electrodes combining the fast charge transport properties of an ultra-thin film and the high optical absorption properties of a thick film. Based on these concepts, we use two ordinary electrochromic polymers, PANI and PolyProDOT-Me_2_, integrated with periodic metallic nanoslit arrays to demonstrate both monochromatic and full-colour fast switching with high optical contrast. Furthermore, the simple and elegant geometry of the proposed configurations can be extended to large areas for mass production on a flexible substrate through techniques such as roll-to-roll nanoimprint lithography^37^,^38^ or nanotransfer printing^39^. Finally, the contrast and speed enhancements observed for the conformal coating of thin electrochromic material can be translated to any optically sensitive material with thickness-dependent properties such as the charge diffusion length, with applications ranging from catalysis to photovoltaics.

## Methods

### Plasmonic electrode preparation

Au and Al films are prepared by direct-current sputtering onto pre-cleaned ultra-flat glass slides coated with a 5-nm-thick Ti adhesion layer. The deposition rate for Au and Al is *R*_Au_≈0.36 nm s^−1^ and *R*_Al_ ≈0.25 nm s^−1^, respectively. For full-colour electrochromic switches, a 170-nm-thick Si_3_N_4_ layer is deposited by sputtering on glass substrate before the deposition of Al film. The deposition rate for Si_3_N_4_ is 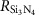 ≈0.15 nm s^−1^. All the depositions are performed at room temperature. Subwavelength slits are prepared by FIB milling using a dual-beam (FIB/SEM) system (Ga^+^ ions, 40 pA beam current, 30 keV beam energy).

### Polymer synthesis and characterization

PANI is synthesized electrochemically from a 2-M HNO_3_ solution containing 15 mM aniline. The films are deposited using potentio-dynamic cycling from −0.2 to 1.05 V versus Ag/AgCl, at a cycling rate of 30 mV s^−1^. A Pt mesh is used as a counter electrode and a Ag/AgCl is used as a reference electrode. Film thickness is controlled by varying the number of cycles (Supplementary Fig. 5). PolyProDOT-Me_2_ is synthesized inside of an Ar glovebox with an Al film from a 10-mM solution of monomer in 0.1 M tetrabutylammonium perchlorate/acetonitrile at a constant potential of 1.3 V versus Ag wire. All films are washed with monomer-free electrolyte solution. Following deposition, polymer-coated electrodes are characterized using cyclic voltammetry in solution consisting of 0.1 mol l^−1^ HNO_3_ and 1 mol l^−1^ NaNO_3_ for PANI and 0.1 mol l^−1^ LiClO_4_ in a mixture (2:1) of dimethyl carbonate and ethylene carbonate for PolyProDOT-Me_2_. Atomic force microscopy (AFM) is used to confirm polymer thicknesses on planar portions of the substrates and scanning electron microscopy is used to image the surface morphology.

### Spectroelectrochemical measurements

All spectroelectrochemical measurements are conducted in custom-built cells consisting of Pt-coated glass electrodes serving as counter electrodes and a micro Ag/AgCl electrode (PANI) or Ag wire (PolyProDOT-Me_2_) used as reference electrodes. All measurements are taken with the same electrolyte solutions used for cyclic voltammetry and in the same cell geometry. In optical transmission experiments, samples are irradiated with a HeNe laser and tungsten halogen bulb, and observed using both upright and inverted optical microscopes. The transmitted light is collected with an amplified photodiode or a spectrophotometer.

## Additional information

**How to cite this article:** Xu, T. *et al.* High-contrast and fast electrochromic switching enabled by plasmonics. *Nat. Commun.* 7:10479 doi: 10.1038/ncomms10479 (2016).

## Supplementary Material

Supplementary Information Supplementary Figures 1 - 5.

Supplementary Movie 1 The Supplementary Movie 1 shows the real-time switching of red light transmitted through the Au-nanoslit electrode coated with PANI film as the applied voltage is repeatedly stepped between VON and VOFF.
