# Supplementary material for: High-contrast and fast electrochromic switching enabled by plasmonics
Source: Nat Commun. 2016 Jan 27;7:10479. doi: 10.1038/ncomms10479 (PMC4737852; doi:10.1038/ncomms10479)
Supplement: Supplementary Information — Supplementary Figures 1 - 5. [file ncomms10479-s1.pdf]

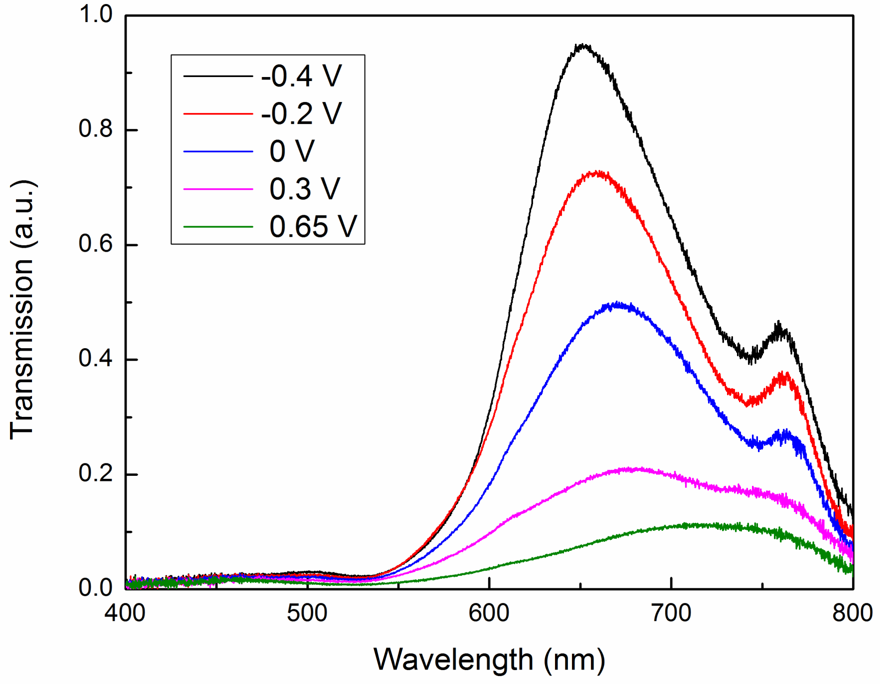

Supplementary Figure 1. Transmission spectrum of a 15 nm-thick PANI film deposited on the Au-nanoslit array, with external applied voltage ranging from – 0.4 V to 0.65 V.

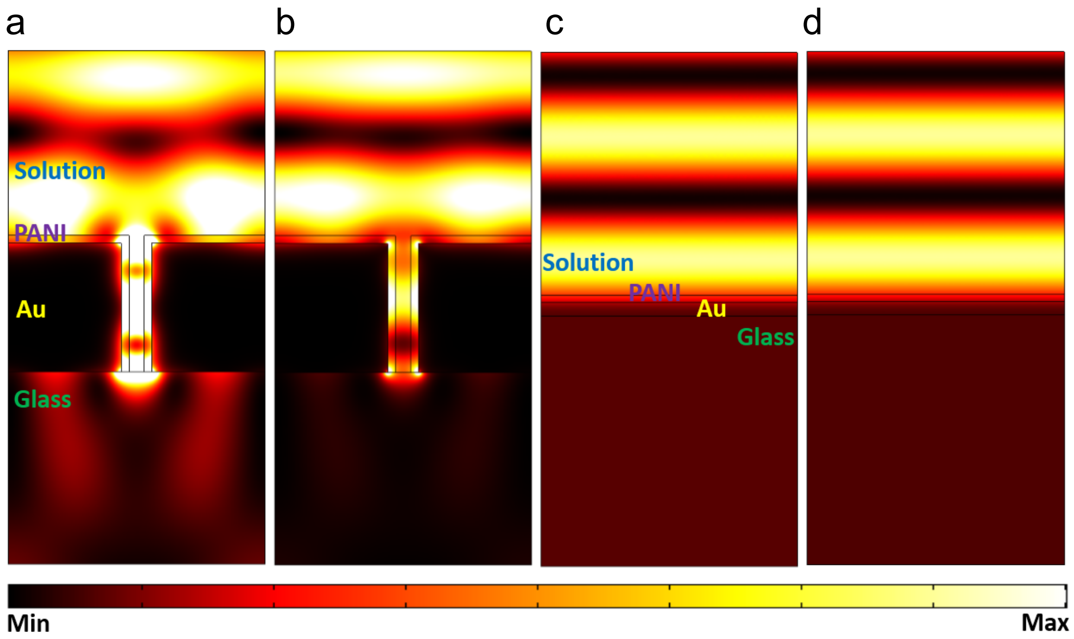

Supplementary Figure 2. FDTD simulated electric field intensities in the Au-nanoslit and reference flat electrochromic devices. The operation wavelength is at 632.8 nm and the PANI film thickness is 15 nm. (a) Au-nanoslit device with PANI film in reduced form; (b) Au-nanoslit device with PANI film in oxidized form; (c) Flat device with PANI film in reduced form; (d) Flat device with PANI film in oxidized form.

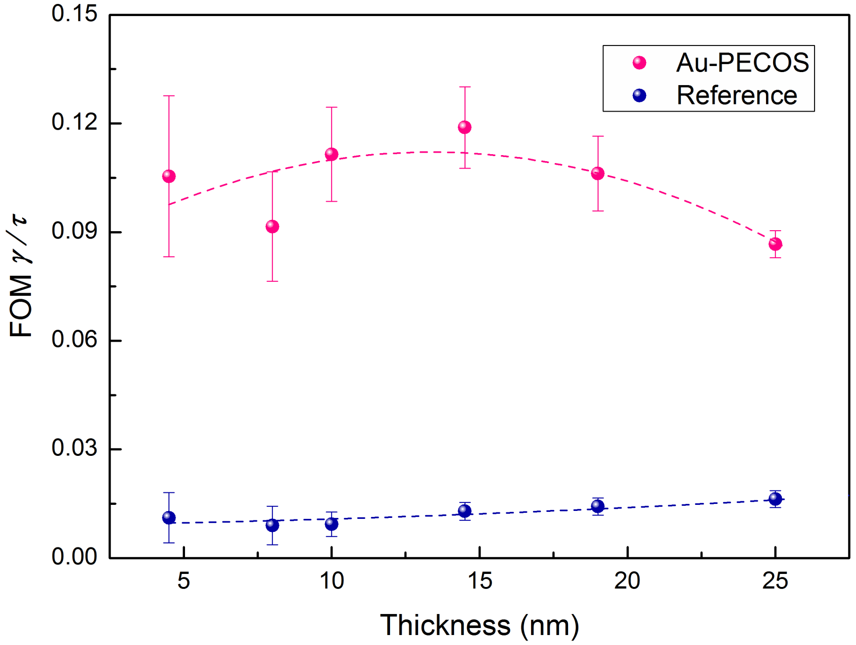

Supplementary Figure 3. Experimental figure of merit (FOM), defined as the ratio between switching contrast γ and time τ, for both Au-nanoslit and reference planar devices. Errors, standard deviation for repeated experimental measurements. The dashed lines indicate a polynomial fit to the data.

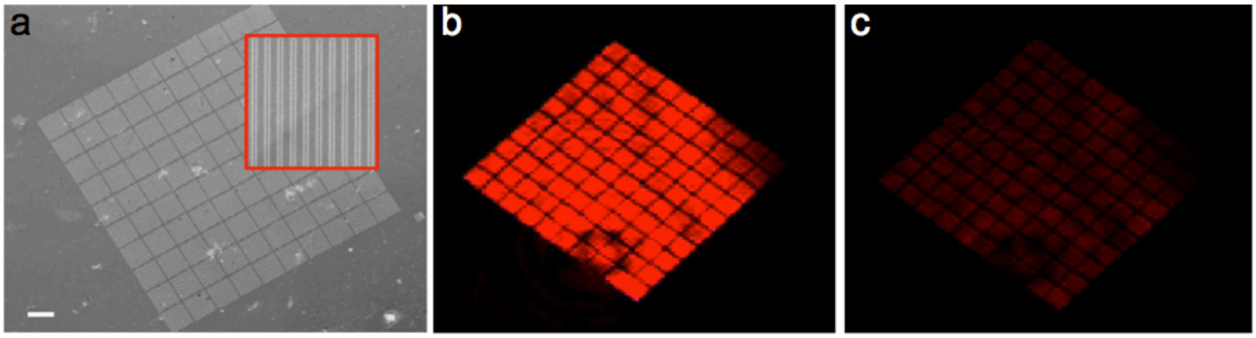

Supplementary Figure 4. (a) Scanning electron micrograph (SEM) image of the large-area Au-nanoslit arrays coated with a 15 nm-thick layer of PANI. Inset shows magnified SEM image. Scale bar: 10um. (b) and (c) Single frame from video of light transmitted through array immersed in a solution of 0.1 M HNO_3 and 1 M $NaNO_3$ and illuminated at λ=633 nm, as the electrochemical cell is switched between (b) -0.2 V and (c) 0.3 V. See video file "**Supplementary Movie 1**" for actual movie.

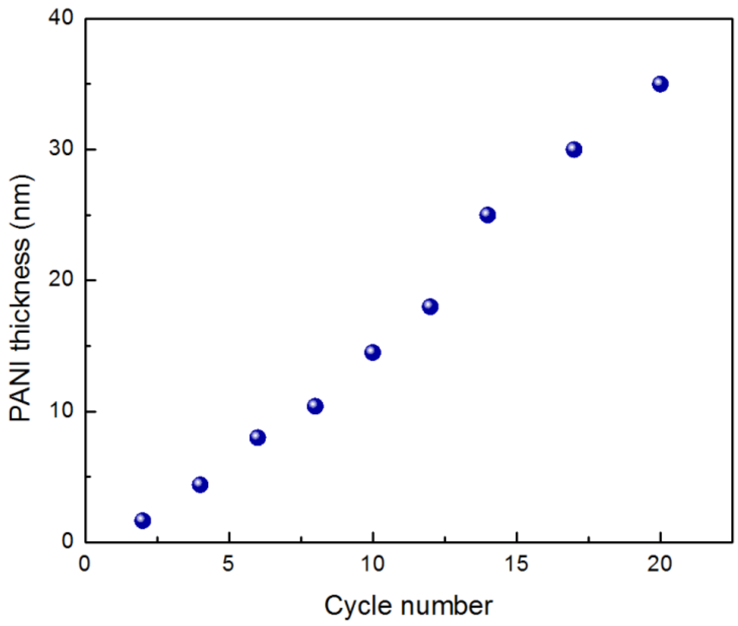

Supplementary Figure 5. Thickness of PANI film deposited on Au electrode as a function of potentio-dynamic electrodeposition cycle number. The film thickness is characterized by atomic force microscopy (AFM).
